# Shark Microbiome Analysis Demonstrates Unique Microbial Communities in Two Distinct Mediterranean Sea Shark Species

**DOI:** 10.3390/microorganisms12030557

**Published:** 2024-03-11

**Authors:** Francesco Montemagno, Chiara Romano, Deborah Bastoni, Angelina Cordone, Olga De Castro, Sergio Stefanni, Emilio Sperone, Donato Giovannelli

**Affiliations:** 1 Department of Biology, University of Naples Federico II, 80126 Naples, Italy; francesco.montemagno@unina.it (F.M.); deborah.bastoni@unina.it (D.B.); olga.decastro@unina.it (O.D.C.); donato.giovannelli@unina.it (D.G.); 2 Department of Biology, Ecology and Earth Sciences, University of Calabria, 87036 Rende, Italy; chiara.romano@unical.it; 3 Botanical Garden, University of Naples Federico II, 80126 Naples, Italy; 4 Stazione Zoologica Anton Dohrn, 80122 Naples, Italy; sergio.stefanni@szn.it; 5 National Research Council, Institute of Marine Biological Resources and Biotechnologies, CNR-IRBIM, 60125 Ancona, Italy; 6 Department of Marine and Coastal Science, Rutgers University, New Brunswick, NJ 08901, USA; 7 Marine Chemistry & Geochemistry Department, Woods Hole Oceanographic Institution, Woods Hole, MA 02543, USA; 8 Earth-Life Science Institute, Tokyo Institute of Technology, Tokyo 152-8550, Japan

**Keywords:** microbiome, shark, Mediterranean Sea, *Somniosus rostratus*, *Prionace glauca*

## Abstract

Our knowledge regarding the role of the microbiome in fish health has been steadily increasing in the last decade, especially for species of commercial interest. Conversely, relatively few studies focus on the microbiomes of wild fish, especially apex predators like sharks, due to lower economic interest and greater difficulty in obtaining samples. Studies investigating microbiome differences between diverse anatomical locations of sharks are limited, and the majority of the available studies are focused on the microbial diversity present on shark teeth, with the aim of preventing infections due to bites of these animals or evaluating the presence of certain pathogens in healthy or diseased specimens. Here, we investigated the skin, mouth, gills, and cloaca microbiomes of five individuals of two phylogenetically distant species of sharks (*Prionace glauca* and *Somniosus rostratus*) to obtain a better understanding of the diversity regarding the microbiomes of these animals, how they change throughout different body parts, and how much they are influenced and determined by the ecology and evolutionary relationship between host and microbiome. To confirm the taxonomy of the sharks under study, we barcoded the specimens by sequencing the mtDNA COI from a biopsy of their skin. Microbial diversity based on the 16S rRNA gene reveals that partially overlapping microbiomes inhabit different body parts of each shark species, while the communities are distinct between the two species. Our results suggest that sharks’ microbiome species-specific differences are controlled by the ecology of the shark species. This is the first study comparatively analyzing the microbiome diversity of different anatomical locations in two shark species of the Mediterranean Sea.

## 1. Introduction

The microbiome is the community of microorganisms that inhabit an organism, establishing a symbiotic relationship with the host. These communities share an intimate bond with their host, being involved in nutrient supplementation, disease susceptibility, stimulating the development of the immune system, and out-competing opportunistic pathogens [1]. Although this symbiotic relationship often benefits the host, it is worth noting that negative interactions also occur within the microbiome. Factors such as environmental stressors, host immune responses, and microbial dysbiosis can lead to disruptions in microbial balance, and potentially harmful outcomes. This symbiotic relationship is also reflected in the lifestyle, ecology, and habitat of the host, and drives patterns and fluctuations in its microbiome [2].

Understanding how environmental changes, ecology, and life history events influence marine animals’ microbiomes is gathering a growing interest within the field of marine science [3]. Although fish account for nearly half of vertebrate species, most of the understanding we have reached about microbiomes and holobionts comes from mammals, which comprise less than 10% of the total vertebrate diversity [4]. A large portion of the studies on marine animal microbiomes are focused on economically significant aquaculture species [5]. Although not completely understood, we know that the microbiome plays a role in influencing the host’s fitness, behavioral and cognitive traits [6], and its overall health through the production of protective secondary metabolites [7] or the modulation of host immunity [3]. In marine animals, the interaction is not only with their own microbiome but also with the one inhabiting their surroundings [5] as these animals share their environment with a vast diversity of microorganisms that play a critical role in oxygen production, nutrient cycling, and organic matter degradation [8,9].

Much of the existing research on fish microbiomes focuses on characterizing the taxonomic diversity of gut microbiomes, and the communities are 90% characterized by members of the phyla Proteobacteria, Bacteroidetes, and Firmicutes [10,11], varying in response to changing nutrition and across other body surfaces, like skin and gills, which is especially interesting in marine animals that are surrounded by the planktonic microbes present in seawater [12] and for which a surface microbiome could play a major role as a protective barrier against pathogens [13]. A factor that strongly influences the microbiomes of fish skin and that is secondarily linked to the host species is skin mucus [14] and the antimicrobial compounds it contains [15], which can act as a buffer against the surrounding environment. Even though strong environmental changes can influence fluctuations in the skin microbiomes of fish, as observed in Atlantic salmon during its migration from rivers to the ocean [16], skin microbiomes remain mostly species-specific [17].

A large portion of our knowledge of fish microbiomes is linked to a strong economic interest in the fishing and aquaculture industry. There is a limited amount of research on the microbiome composition of elasmobranchs [18], especially regarding sharks, linked to lower economic interest and to the difficulty in studying these animals. The few studies that take into account sharks’ microbiomes in different anatomical locations often focus on teeth bacteria, with the aim of preventing infections due to bites of these animals or evaluating the presence of pathogens in healthy vs diseased specimens [18,19]. Only a small fraction of studies are focused on the influence of host ecology, population-level dynamics, or phylogeny regarding the shark microbiome [20,21,22]. Sharks-associated microbial communities can establish symbiotic associations in various anatomical locations, possibly contributing to the defense of the host against pathogens and susceptibility to disease [2]; however, these mechanisms in sharks are still poorly understood. Studying the microbiomes of these animals is not only important to improve our knowledge about these species and to use them as ecological indicators of ecosystem status but also to characterize new microbial diversity and gain more information on shark bite treatment protocols.

Studying the symbiotic microbial communities of an organism can provide crucial information to understand the natural history, ecology, and main processes required for the function and survival of their hosts. Even though sharks are widespread animals, their microbial communities still remain poorly understood, both because of the difficulties in studying sharks in the field and because they have lower commercial value compared to bony fish, making less funding available for research.

Here, we provide information on the microbiome diversity in two different species of sharks to obtain a better understanding of the microbiomes of these animals, how they change throughout different body parts, and how much they are influenced and determined by the ecology and phylogeny of the host. In spite of the limited number of animals and samples collected, reflecting the difficulty in obtaining such samples, this study provides valuable baseline data that will contribute to understanding sharks’ microbiome diversity, a field largely unexplored to date. Studies like this could provide information about how much the habitat, diet, and ecology of a species can influence and explain microbiome variations and provide specific information on how the gender, age, and habitat of a single individual can shape diversity. Ecological processes and environmental factors could explain some of the variations in the microbiomes of different sharks, but including sharks’ phylogeny and population structure will help us also to take into account evolutionary and coevolutionary processes that have shaped the meta-organism.

## 2. Materials and Methods

### 2.1. Sample Collection

Five individuals of two shark species were caught in Mar Ionio between August 2020 and September 2020 and released after measurements and sample collection. Handling time was kept to a minimum and all sharks were released in as little time as possible to ensure survival and low stress due to the catch and handling.

The sampling allowed us to collect samples from different anatomical locations from a total of five shark specimens (Table 1). For each shark, morphometric information (species, sex, length, weight, and sexual maturity), four swabs, a skin sample, and an environmental sample (seawater sample) were collected. The skin samples for the determination of shark species through molecular approaches were taken from the posterior profile of the dorsal fin, in the junction between the fin and the back of the animal, as it is an area with little exposure to the sea current.

Gills, mouth, skin, and cloaca samples of each individual were taken using sterile swabs for microbiome analysis. The skin swabs were sampled in the same location as the skin samples, swiping the swab front to back and rotating it four times. For the cloaca samples, the swabs were inserted in the cloacal fissure and rotated. The gill swabs were taken from the third gill and sampled starting from the head, sliding the swab once in a dorsal-ventral direction and once in a ventro-dorsal direction. The last swab, taken in the mouth, was collected by placing the swab at the lateral attachment of the mouth, directing it once towards the upper gum and returning to the attachment and once turning towards the lower gum and returning to the attachment. All swabs were kept cool after sampling and once on the ground frozen at −20 °C until DNA extraction. At the sampling site of each shark, a 3 L water sample was collected, filtered using a Sterivex 0.22 µm filter (Merck Millipore, Milan, Italy), and stored in the freezer at −20 °C. Among the collected samples, FILTER-AD and SWAB-6 samples were excluded from the downstream analysis due to inadequate sequencing depth (Table 1).

### 2.2. DNA Extraction and Sequencing

DNA was extracted from the skin samples using a modified phenol–chloroform extraction protocol [23,24] and used for the shark species identification. DNA visualization was performed by agarose gel electrophoresis, and DNA quantification was determined by NanoDrop spectrophotometer (Thermo Fisher Scientific, Roma, Italy).

A Polymerase Chain Reaction [25] was performed using 2X Phire Plant Direct PCR Master Mix (Thermo Fisher Scientific, Italy), ElasmoCR15642F (5′-TTG GCT CCC AAA GCC AAR ATT CTG-3′;) and ElasmoCR16638R (5′-CCC TCG TTT TWG GGG TTT TTC GAG-3′) as primer for the CR (Control Region) [26,27], while using VF2 -t1 (5′-TCA ACC AAC CAC AAA GAC ATT GGC AC-3′) and FR1d-t1 (5′-CAC CTC AGG GTG TCC GAA RAA YCA RAA-3′) as primer for the COI (Cytochrome c oxidase I) [28]. The cycling parameters were performed according to the manufacturer’s instructions.

The amplicons were purified using the Monarch^®^ PCR & DNA Cleanup Kit (New England Biolabs, County Road, Ipswich, MA, USA) and sequenced and analyzed as previously reported [29].

Microbiome DNA extraction was performed on the swabs and on the filtered water samples using the DNeasy PowerSoil Kit (QIAGEN, Milano, Italy) following the manufacturer’s instruction with some minor modifications. In particular, the Elution was carried out by passing twice the same volume (50 µL) of solution C6 to collect the DNA. DNA visualization was performed by agarose gel electrophoresis and visualized with an ultraviolet transilluminator (ChemiDoc™ XRS, Bio-Rad, Segrate, Italy). DNA quantification was determined by the NanoDrop spectrophotometer. The obtained DNA was sequenced at the Integrated Microbiome Resources (https://imr.bio/, accessed on 8 January 2023) using primers targeting the V6–V8 region of the 16S rRNA (B969F = ACGCGHNRAACCTTACC and BA1406R = ACGGGCRGTGWGTRCAA) using Illumina Miseq technology.

### 2.3. Statistical Analysis

The microbiome sequences obtained were analyzed using the DADA2 package [30] after removing primers and adapters. All sequences with an average call quality for each base between 20 and 40 were retained for downstream processing. DADA2’s end product was an amplicon sequence variant (ASV) table, a higher-resolution version of an out table, and the Silva database release 138 was used as a comparison to assign the taxonomy to the sequence variants (https://www.arb-silva.de/, accessed on 1 June 2023). The end product of DADA2 was used to investigate the prokaryotic diversity using the phyloseq package as previously described [31,32,33].

The alpha diversity was calculated using the Shannon diversity index, and a Kruskal–Wallis rank test [34] was used to test any significant difference in alpha diversity between shark species or between anatomical locations. The beta diversity of the community was investigated using weighted and unweighted Jaccard dissimilarity index and related to environmental factors using non-metric multidimensional scaling (nMDS) with the vegan package [35]. Significance tests were performed using permutational multivariate analysis of variance (PERMANOVA) through the Adonis function.

Sharks’ COI and CR sequences and microbiome sequences analyzed in this study are available through the European Nucleotide Archive (ENA) under project accession PRJEB60929. A complete R script containing all the steps to reproduce our analysis is available at https://github.com/giovannellilab/Montemagno_et_al_Shark_Microbiome, accessed on 30 June 2023, and with DOI https://doi.org/10.5281/zenodo.7584581, accessed on 30 June 2023 together with all the environmental data.

## 3. Results

### 3.1. Shark and Seawater Microbial Community Diversity

The results of molecular analysis of sharks’ COI and CR sequences have confirmed that the species under study are Prionace glauca and Somniosus rostratus (Table 1). The 16S rRNA tag-amplicon sequencing of 19 samples (gills, mouth, skin, and cloaca from each individual) from five sharks and three water samples produced a total of 1,431,719 high-quality 16S rRNA reads after quality check and merging (Table 2), and they were used to identify 803 unique ASVs. After taxonomic annotation, we were able to classify 58.65% of the ASVs at the genus level.

**Figure 1:**
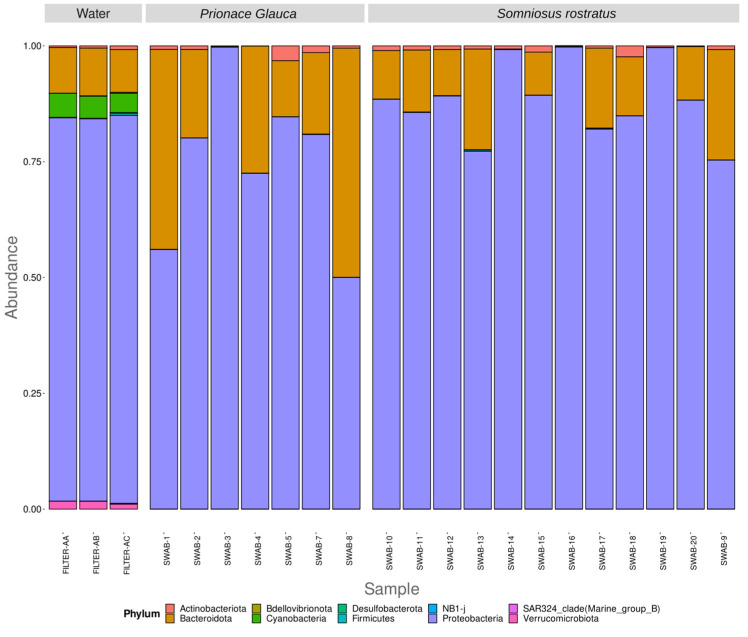
Phylum-level distribution of the 16S rRNA diversity among samples. Relative abundance reported as 1 = 100% of the total reads.

**Figure 2:**
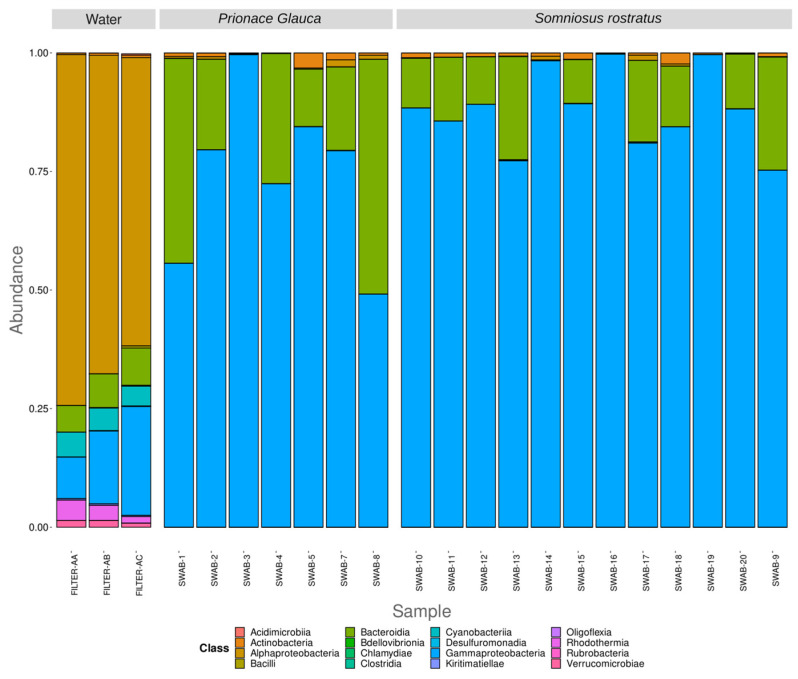
Class-level distribution of the 16S rRNA diversity among the samples. Relative abundance reported as 1 = 100% of the total reads.

Two samples, Filter-AD and Swab-6, were excluded from the present analysis due to inadequate sequencing depth. In total, ten prokaryotic phyla have been identified. Sequences relative to the phyla *Proteobacteria*, *Bacteroidota*, *Actinobacteriota*, and *Firmicutes*, with an average relative abundance of 83.27%, 14.96%, 1.28%, and 0.07%, respectively, are shared between the water and shark samples. While there are no unique phyla for the shark samples, the eight phyla *Cyanobacteria*, *Verrucomicrobia*, *Bdellovibrionota*, *SAR324-clade* (Marine group B), *Desulfobacterota*, and *NB1-j* are only found in water samples, with average relative abundance values of 4.74%, 1.51%, 0.09%, 0.07%, 0.06%, and 0.01%, respectively. At the phylum level, water and shark samples show a distinct microbial community, while there are no remarkable differences in terms of microbial community at the phylum level between the two shark species or between the different anatomical locations investigated (Figure 1). *Gammaproteobacteria*, *Bacteroida*, and *Alphaproteobacteria* are the most abundant classes (73.81%, 14.55%, and 9.46%, respectively) shared between sea water and shark microbial communities. The classes *Actinobacteria* and *Bacilli* are still shared between the two types of samples, although with lower abundances. *Rhodothermia*, *Cyanobacteria*, *Verrucomicrobiae*, *Kiritimatiellae*, *Acidimicrobiia*, *Bdellovibrionia*, *Desulfuromonadia*, *Chlamydiae*, and *Oligoflexia* are the classes only found in the water samples. The *Clostridia* class is only present in two skin shark samples, both belonging to a *Somniosus rostratus* species, while the *Rubrobacteria* class is only present in one *S. rostratus* skin sample (Figure 2).

**Figure 3:**
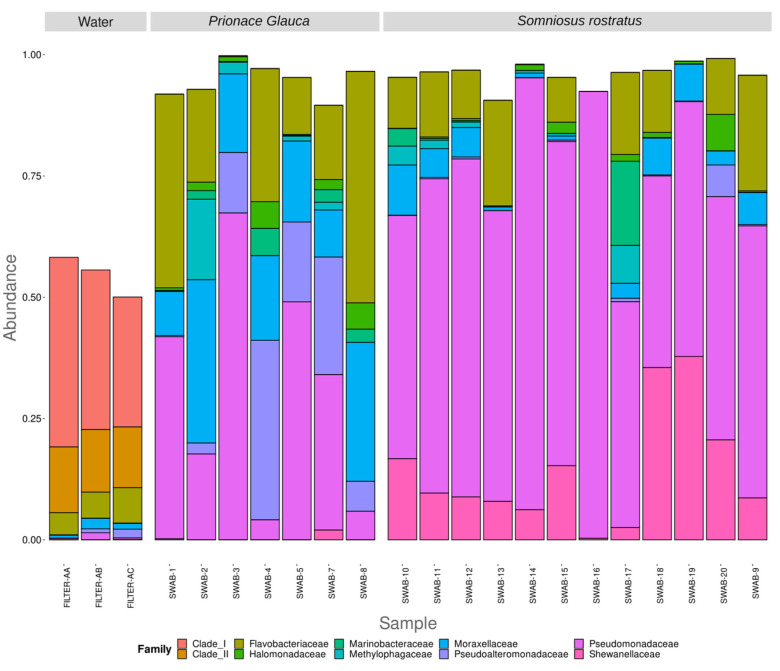
Family-level distribution of the 16S rRNA diversity among the samples. Only the top 10 most abundant families are reported. Relative abundance reported as 1 = 100% of the total reads.

The ten most abundant families are Pseudomonadaceae, Moraxellaceae, Shewanellaceae, Flavobacteriaceae, Clade-I, Pseudoalteromonadaceae, Marinobacteraceae, Methylophagaceae, Halomonadaceae, and Clade-II, with Clade-I and Clade-II being only present in the sea water communities, while Marinobacteraceae, Methylophagaceae, and Halomonadaceae only in shark microbiomes (Figure 3). At the family level, differences between the two shark species are visible in terms of microbial community composition. Between the less abundant families, the Yersiniaceae family is present in all the Somniosus rostratus samples while being completely absent in Prionace glauca, opposite to the Erwiniaceae family, which appears only in Prionace glauca (Supplementary Table S1). Looking at the relative abundance of the shared families, we see other differences between the two shark species. The Somniosus rostratus species shows a noticeably higher relative abundance of families like Pseudomonadaceae and Shewanellaceae (61.45% and 14.16%) if compared to Prionace glauca (31.13% and 0.32%), while the Moraxellaceae family is much more abundant in Prionace glauca (18.79%) than in Somniosus rostratus (4.40%) (Figure 3). There are no evident differences between the microbiome compositions of the different anatomical locations sampled. 

### 3.2. Differences in Microbiome between Environmental and Biological Samples

**Figure 4:**
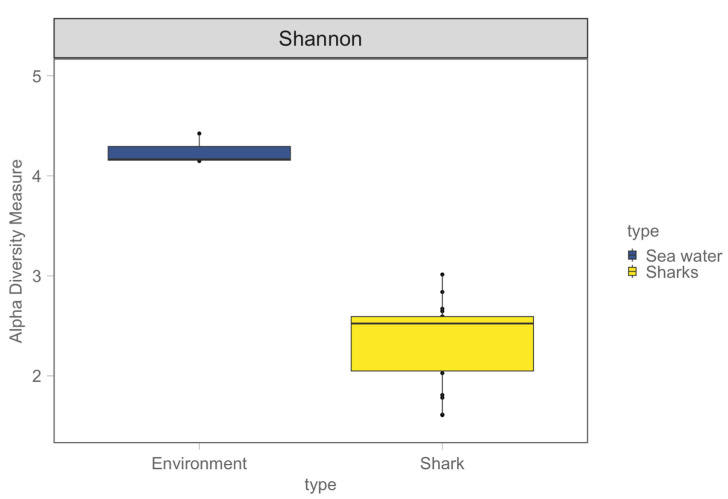
Shannon alpha diversity index for shark microbiome samples and water samples, with box plots displaying the median (bold line), 25th–75th percentiles.

**Figure 5:**
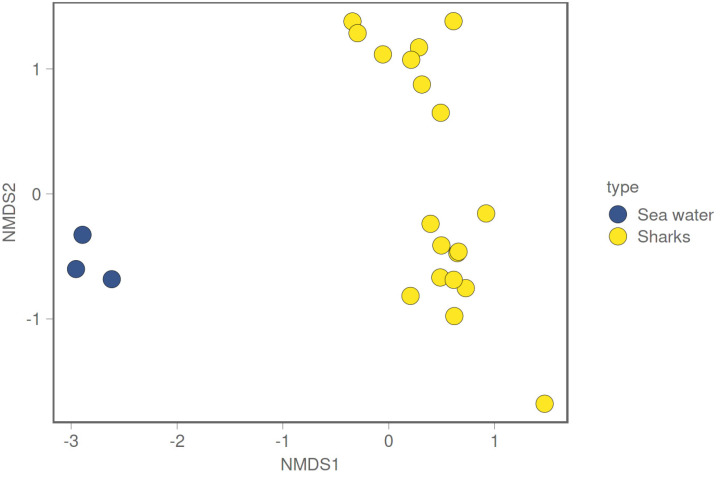
Weighted non-metric dimensional scaling based on the Jaccard similarity index of sharks and water samples.

Shark microbiome samples appear significantly different from the seawater microbiome in both alpha and beta diversity (Figure 4 and Figure 5). This result is confirmed by a Kruskal–Wallis test performed on the Shannon alpha diversity index (Kruskal–Wallis, chi-squared = 7.4348, df = 1, *p*-value = 0.006398) and an Adonis test confirming a significant difference between the two groups in weighted Jaccard non-metric dimensional scaling (Figure 5) (Adonis test, *p* value = 0.024). As clearly shown in Figure 5, shark samples and sea water samples appear in two distinct clusters; this result does not exclude the microbial communities living the in water samples, and those found on the shark body do influence each other in such a way.

### 3.3. Different Microbiomes between the Two Shark Species and between Anatomical Locations

**Figure 6:**
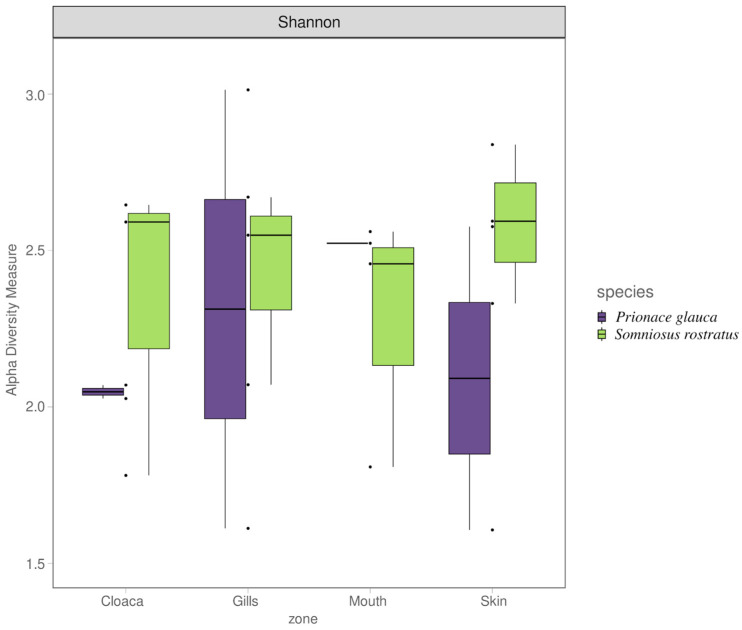
Shannon alpha diversity index for shark microbiome samples across anatomical locations, colored for the shark species.

The Shannon diversity index was used to investigate the alpha diversity between the two shark species, grouping the anatomical location for each one. As reported in Figure 6, Somniosus rostratus microbiome samples show an overall higher alpha diversity, but a Kruskal–Wallis test showed no significant differences amongst the different anatomical locations (Kruskal–Wallis, chi-squared = 0.81, df = 1, *p*-value = 0.8471).

**Figure 7:**
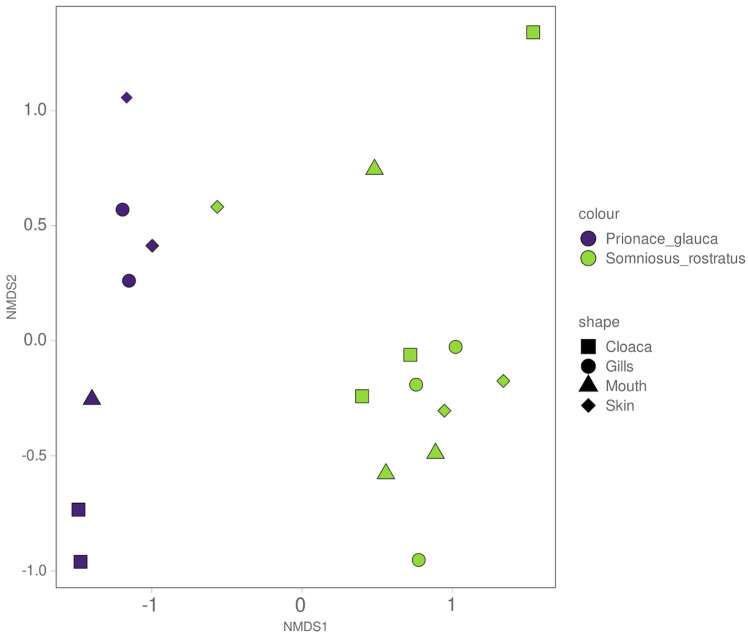
Weighted non-metric dimensional scaling based on the Jaccard similarity index colored for the two species and shaped by the anatomical locations.

**Figure 8:**
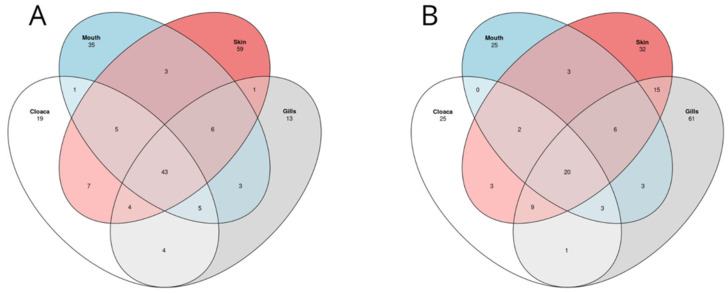
Venn diagram showing shared and unique ASVs across the anatomical location for *S. rostratus* (**A**) and *P. glauca* (**B**). For the analysis, we used 208 ASVs, 11 samples for *S. rostratus* and 8 samples for *P. glauca*.

The beta diversity of the shark microbiome was investigated by removing the water samples. Weighted non-metric dimensional scaling based on the Jaccard similarity index (Figure 7) shows good separation between the two shark species, while there is no visible trend in the distribution of the different anatomical locations. These results were confirmed by an Adonis test, which confirmed a significant difference in microbial community between the two species (Adonis test, *p* value = 0.0001) and no significant difference between the different anatomical locations (Adonis test, *p* value = 0.785). To better define the core microbiome of each shark species, we used a Venn diagram to visualize the shared and unique ASVs across the different anatomical locations. As shown in Figure 8, we identified 43 (20.7%) and 20 (9.6%) ASVs out of 208 total ASVs in the core microbiomes of *S. rostratus* and *P. glauca*, respectively. In both shark species, the most abundant shared ASVs at the phylum level were Proteobacteria (94% in *S. rostratus* and 82.21% in *P. glauca*), Bacteroidota (4.93% in *S. rostratus* and 16.67% in *P. glauca*), and Actinobacteria (0.87% in *S. rostratus* and 1.12% in *P. glauca*). We also found Firmicutes at a lower percentage (0.017%) only in the core microbiome of *S. rostratus.*

## 4. Discussion

Agreeing with previous studies that investigate shark microbiomes and their surrounding non-symbiont communities [2,36], our study confirms that shark-associated microbial communities are significantly different from the microbiomes of the surrounding environment regarding both richness and diversity. Our results show an overlap of only a number of microbial taxa between the shark microbiome and the microbial community of the surrounding seawater. Consistent with the results obtained by Karns (2017) [36], water microbial communities have significantly higher alpha diversity values when compared to shark ones, but, when looking at the beta diversity for both types of samples, shark microbial communities are much more variable than water samples. Our results agree with studies conducted on other marine organisms like fish and dolphins [21,37,38], showing that the main driver that affects sharks’ microbial community is the specific host and the species of belonging. We found that the overall microbial community composition differs significantly between shark species (*Prionace glauca* and *Somniosus rostratus*), between shark and environmental samples, and that sharks’ microbiomes do not differ significantly between different anatomical locations in both alpha and beta diversity.

Species-specific microbiomes have already been observed in different shark species [18,21], and in other marine predators like sperm whales (*Physeter macrocephalus*; Erwin et al., 2017) and killer whales (*Orcinus orca*) [37,38]. A coevolution of the host and its associated microbiome has been demonstrated by previous studies [39,40,41], suggesting the influence of the host ecology on its microbiome. Moreover, a number of studies have demonstrated how environmental conditions such as pH, salinity, and temperature can have an influence on the host-associated microbiota [42,43,44]. According to these findings, our results suggest that shark species feeding ecologies, migration patterns, and many other ecological parameters could influence specialized microbial communities. As mentioned by Storo et al., 2021 [21], shark changes in depth during migrations and parturition could influence their microbiome composition. Moreover, Pogoreutz and collaborators [19] found differences in the skin microbial community composition of blacktip reef sharks (*Carcharhinus melanopterus*, [45]), linked by the geographic area. *Somniosus rostratus* is known to live in a depth range between 200 and 2200 m [46], while *Prionace Glauca* lives up to 600 m in depth [47]. Such differences in habitat and behavior, and therefore in diet, could cause differences in these species’ microbiomes. 

Other studies focused on shark-associated microbiomes took into account different anatomical locations (gills, teeth, skin, and cloaca) [18,21,48]. Differently from these studies, we were not able to detect any significant difference between the anatomical locations taken into account for both species. A larger dataset, including either more shark species or a larger number of individuals belonging to the same species, could help to elucidate in a clearer way differences in microbial community composition across anatomical locations not visible in the current study and also help to determine the weight of ecology and phylogeny on the composition of the microbiome. We also investigated if other variables such as age, size, and sex had an influence on microbiome composition, but none of them showed a significant influence.

Previous studies describing sharks’ bacterial community include other shark species and other anatomical locations, and only a few have been conducted in the Mediterranean Sea, making comparison difficult. Nonetheless, we are still able to find a number of bacterial taxonomic groups already described in previous studies on sharks’ microbiomes. Our study identifies a core microbiome shared between the two shark species taken into account and a small number of bacterial groups that could be species-specific.

The *Flavobacteriaceae* family is one of the most abundant families in our samples. This family has already been found in the gut and skin microbiomes of different marine animals [49,50] and in a wide range of habitats, like marine and freshwater [51]. Banning et al. [52] suggested a bacteriolytic role of this family, which could contribute to the health of the host by warding off pathogenic bacteria. In particular, the *Lacinutrix* genus represents the largest portion of the *Flavobacteriaceae* sequences found in our samples, a genus found in the microbiomes of jellyfish, algae, and in marine sediments [50,53,54].

The genera *Pseudoalteromonas, Psychrobacter*, and *Pseudomonas* were three of the most abundant taxa in our sharks’ microbiomes, and they are also reported by other studies describing the thresher shark (*A. vulpinus*) [2], blacktip reef shark (*C. melanopterus*) [2,19], dusky shark (*Carcharhinus obscurus*), and sandbar shark (*Carcharhinus plumbeus*) [55] microbiomes. 

The genus Pseudoalteromonas has been reported as a common component of sharks’ skin microbiomes, like in the common thresher shark, the lemon shark, and the nurse shark [2,56]. This genus is known for encoding gene functions that may promote healthy microbiome–host interactions [57]. *Pseudoalteromonas* is known for synthesizing antimicrobial compounds that could promote a healthy microbiome and hinder the settlement of eukaryotic marine fouling organisms [57]. Different studies have suggested that *Pseudoalteromonas*, together with the genus *Marinobacter*, also abundant in our sharks, can contribute to the production of antimicrobial compounds able to prevent biofouling and molecules (e.g., lipopolysaccharide, LPS) and able to mediate the host inflammatory response, respectively [57,58,59].

One other bacterial genus that has been linked with healthy hosts is *Psychrobacter,* abundant in our microbiome samples and also reported in the skin mucus of bony fish [60] and other marine organisms like algae, sponges, humpback whale, other marine mammals, and blacktip reef sharks [18,19,49,61,62].

Among the genera known to be present in marine organisms’ microbiomes [63,64] or as pathogens [65,66], there are *Photobacterium* and *Vibrio*, both present in our samples. Different studies have reported the presence of *Photobacterium* in sharks’ gastrointestinal tracts (i.e., gut and cloaca) [67,68,69,70], suggesting it as a common genus of sharks’ core intestinal microbiome [48].

## 5. Conclusions

Our study evaluates possible differences in the microbiomes of different anatomical locations belonging to two shark species from the Mediterranean Sea, with the aim of investigating the microbiomes of these animals. As far as we know, this is the first study focused on the microbiome composition of different shark species and anatomical locations performed in the Mediterranean Sea. The 16S rRNA microbial diversity analysis allowed us to confirm that, as reported by other studies, shark-associated microbial communities are significantly different between each species. Moreover, the differences associated with the different anatomical locations do not follow a predictable trend, and shark microbiomes are distinct from the surrounding ones. Our findings are consistent with previous studies on fish, sharks, and rays showing dominance of members of the Actinobacteria, Proteobacteria, and Bacteroidota phyla. Despite the limitations posed by a comparatively small cohort of animals and collected samples, we believe that our research will serve as a valuable contribution to unravelling the complexity of sharks’ microbiomes by shedding light on the intricate relationship between sharks and their microbial communities, a research area that remains largely unexplored.

## Figures and Tables

**Table 1:** Number and characteristics of the different collected samples.

Code	Individual	Species	Length (cm)	Weight (kg)	Water Temp (°C)	Air Temp	Sex	Type	Month	Zone
FILTER-AA	ind_01	-	NA	NA	27 °C	34 °C	-	Environment	Aug	Water
FILTER-AB	ind_02	-	NA	NA	26 °C	30 °C	-	Environment	Sept	Water
FILTER-AC	ind_03/ ind_04	-	NA	NA	28 °C	28 °C	-	Environment	Sept	Water
FILTER-AD	ind_05	-	NA	NA	28 °C	26 °C		Environment	Sept	Water
SWAB-1	ind_01	*Prionace glauca*	100 cm	4 kg	27 °C	34 °C	F	Shark	Aug	Skin
SWAB-10	ind_03	*Somniosus rostratus*	100 cm	500 g	28 °C	28 °C	F	Shark	Sept	Mouth
SWAB-11	ind_03	*Somniosus rostratus*	100 cm	500 g	28 °C	28 °C	F	Shark	Sept	Gills
SWAB-12	ind_03	*Somniosus rostratus*	100 cm	500 g	28 °C	28 °C	F	Shark	Sept	Cloaca
SWAB-13	ind_04	*Somniosus rostratus*	110 cm	600 g	28 °C	28 °C	M	Shark	Sept	Skin
SWAB-14	ind_04	*Somniosus rostratus*	110 cm	600 g	28 °C	28 °C	M	Shark	Sept	Mouth
SWAB-15	ind_04	*Somniosus rostratus*	110 cm	600 g	28 °C	28 °C	M	Shark	Sept	Gills
SWAB-16	ind_04	*Somniosus rostratus*	110 cm	600 g	28 °C	28 °C	M	Shark	Sept	Cloaca
SWAB-17	ind_05	*Somniosus rostratus*	120 cm	700 g	28 °C	25 °C	F	Shark	Sept	Skin
SWAB-18	ind_05	*Somniosus rostratus*	120 cm	700 g	28 °C	25 °C	F	Shark	Sept	Mouth
SWAB-19	ind_05	*Somniosus rostratus*	120 cm	700 g	28 °C	25 °C	F	Shark	Sept	Gills
SWAB-2	ind_01	*Prionace glauca*	100 cm	4kg	27 °C	34 °C	F	Shark	Aug	Mouth
SWAB-20	ind_05	*Somniosus rostratus*	120 cm	700g	28 °C	25 °C	F	Shark	Sept	Cloaca
SWAB-3	ind_01	*Prionace glauca*	100 cm	4 kg	27 °C	34 °C	F	Shark	Aug	Gills
SWAB-4	ind_01	*Prionace glauca*	100 cm	4 kg	27 °C	34 °C	F	Shark	Aug	Cloaca
SWAB-5	ind_02	*Prionace glauca*	100 cm	4 kg	26 °C	30 °C	M	Shark	Sept	Skin
SWAB-6	ind_02	*Prionace glauca*	100 cm	4 kg	26 °C	30 °C	M	Shark	Sept	Mouth
SWAB-7	ind_02	*Prionace glauca*	100 cm	4 kg	26 °C	30 °C	M	Shark	Sept	Gills
SWAB-8	ind_02	*Prionace glauca*	100 cm	4 kg	26 °C	30 °C	M	Shark	Sept	Cloaca
SWAB-9	ind_03	*Somniosus rostratus*	100 cm	500 g	28 °C	28 °C	F	Shark	Sept	Skin

**Table 2:** Number of reads at different steps of the analysis, together with the number of obtained ASVs per sample.

Sample	Input	Filtered	Merged	ASVs
FILTER-AA	112,008	101,664	99,887	85,808
FILTER-AB	69,153	62,781	61,682	53,766
FILTER-AC	19,029	17,085	16,396	13,940
SWAB-1	63,583	58,098	57,240	45,926
SWAB-10	70,926	64,838	64,219	49,732
SWAB-11	58,219	52,296	51,836	45,801
SWAB-12	99,394	90,943	90,022	69,005
SWAB-13	131,764	121,383	120,551	101,330
SWAB-14	106,950	97,336	96,756	80,236
SWAB-15	85,444	78,023	77,420	61,340
SWAB-16	39,479	36,021	35,781	34,578
SWAB-17	58,712	54,295	53,275	43,558
SWAB-18	77,510	71,540	70,959	60,912
SWAB-19	73,355	68,062	67,751	64,847
SWAB-2	59,285	54,880	54,104	41,789
SWAB-20	111,213	103,042	101,995	76,030
SWAB-3	47,398	43,992	43,627	37,508
SWAB-4	78,454	73,440	72,583	61,472
SWAB-5	70,298	65,096	64,539	58,471
SWAB-7	67,555	61,574	60,268	36,328
SWAB-8	35,618	33,178	32,551	27,185
SWAB-9	42,449	38,695	38,277	28,961

## Data Availability

All the sequences analyzed in this study are available through the European Nucleotide Archive (ENA) under project accession PRJEB60929. A complete R script containing all the steps to reproduce our analysis is available at https://github.com/giovannellilab/Montemagno_et_al_Shark_Microbiome with DOI https://doi.org/10.5281/zenodo.7584581 together with all the environmental data, accessed on 30 June 2023.

## Supplementary Materials

The following supporting information can be downloaded at: https://www.mdpi.com/article/10.3390/microorganisms12030557/s1, Table S1: summary_barplot.
